# Complex Relationships between HIV-1 Integrase and Its Cellular Partners

**DOI:** 10.3390/ijms232012341

**Published:** 2022-10-15

**Authors:** Anna Rozina, Andrey Anisenko, Tatiana Kikhai, Maria Silkina, Marina Gottikh

**Affiliations:** 1 Faculty of Bioengineering and Bioinformatics, Lomonosov Moscow State University, 119992 Moscow, Russia; 2 Chemistry Department, Lomonosov Moscow State University, 119992 Moscow, Russia; 3 Belozersky Institute of Physico-Chemical Biology, Lomonosov Moscow State University, 119992 Moscow, Russia

**Keywords:** HIV-1, integrase, cellular partners

## Abstract

RNA viruses, in pursuit of genome miniaturization, tend to employ cellular proteins to facilitate their replication. HIV-1, one of the most well-studied retroviruses, is not an exception. There is numerous evidence that the exploitation of cellular machinery relies on nucleic acid-protein and protein-protein interactions. Apart from Vpr, Vif, and Nef proteins that are known to regulate cellular functioning via interaction with cell components, another viral protein, integrase, appears to be crucial for proper virus-cell dialog at different stages of the viral life cycle. The goal of this review is to summarize and systematize existing data on known cellular partners of HIV-1 integrase and their role in the HIV-1 life cycle.

## 1. Introduction

**Figure 1:**
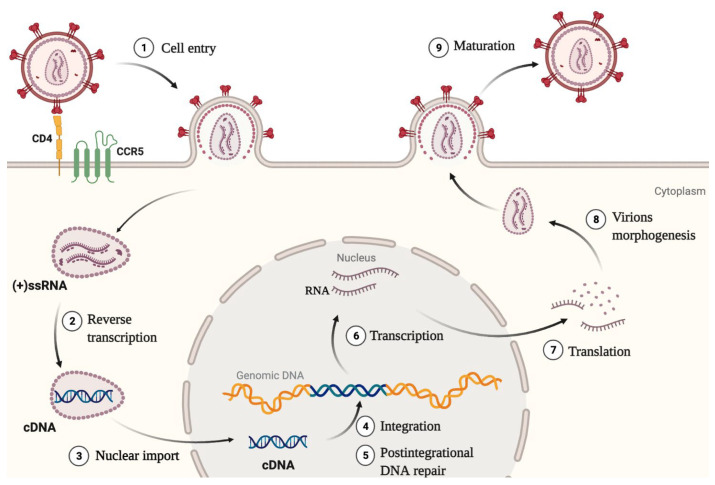
Schematic representation of HIV-1 life cycle. The early replicative stages include binding of the virion to the CD4 receptor and fusogenic co-receptor (CCR5 or CXCR4), cell entry, reverse transcription that induces the subsequent capsid rearrangement and uncoating of PIC before arriving at the nuclear pore, at the nuclear pore complex during the nuclear import of PIC or inside the nucleus (see Section 6), cDNA integration into actively transcribed regions of the cellular genome, and post-integration DNA repair that results in the provirus formation. The late replicative stages include transcription of the viral full-length genomic RNA (gRNA), formation of partially and fully spliced viral mRNA, export of viral gRNA and mRNA, protein synthesis, virion assembly, budding, and maturation.

The HIV-1 life cycle is typical for retroviruses and is schematically represented in Figure 1. It can be divided into early and late stages. Early stages aim at forming a provirus, i.e., a DNA copy of the viral RNA integrated into the cellular genome. After entering the cell, the viral RNA genome enters the cytoplasm, where reverse transcription starts. HIV-1 ssRNA and then its synthesized cDNA interact with a number of cellular and viral proteins, forming a dynamic structure called preintegration complex, or PIC. Viral cDNA is imported into the nucleus, where integration and postintegrational DNA repair take place to form a provirus. Viral cDNA is also found in cells in unintegrated forms, linear or two types of circular 1-LTR and 2-LTR episomal DNA [1]. Late stages of the HIV-1 life cycle following provirus formation include transcription and splicing of viral genomic RNA, viral RNA export through the nuclear pore, polyprotein synthesis, viral particle morphogenesis, and budding of new immature virions. For a virus to become infectious, the viral protease must cleave the Gag and Gag-Pol polyproteins into functional proteins. Late stages result in the production of new viral particles [2].

During the HIV-1 life cycle, viral components interact with numerous cellular proteins. The latter can suppress or assist HIV-1 replication, and in these cases, they are called negative (or restriction) or positive (or co-) factors of viral replication [3]. For one of the viral proteins, called integrase (IN), the largest number of cellular proteins that interact with IN and thus regulate the HIV life cycle have been identified and will be discussed in this review.

HIV-1 IN is one of the three viral enzymes. It belongs to the polynucleotidyltransferase superfamily, which also includes other integrases, transposases, RuvC resolvase, ribonuclease H, and other proteins [4]. Polynucleotidyltransferases catalyze the transfer of nucleic acids, in the case of IN—the transfer of the viral cDNA into the genome of the host cell. To do this, IN in complex with cDNA and other viral and cellular proteins (PIC) catalyzes the cleavage of dinucleotides from both 3′-ends of the cDNA (this process is called 3′-processing), binds cellular target DNA and carries out the strand transfer reaction, which is the insertion of each of the processed viral DNA 3′-ends into each strand of cellular DNA [5].

**Figure 2:**
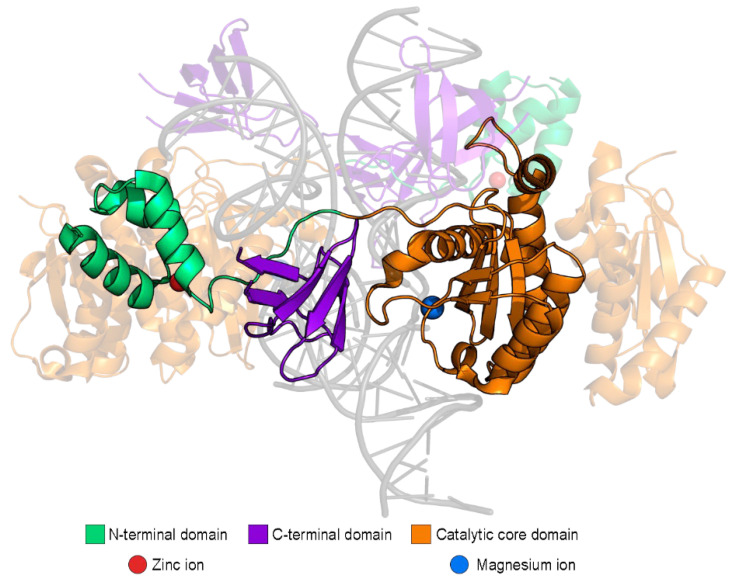
Structure of integrase domains within the HIV-1 intasome (PDB ID 5U1C).

HIV-1 IN consists of three domains: N-terminal, catalytic core, and C-terminal domain, each of which is required for the integration process (Figure 2) [6,7]. The catalytic core domain is the most stable and conservative of the three. It contains an active center that performs both 3′-processing and strand transfer reactions. In addition, this domain has a nuclear localization signal that is thought to contribute to the nuclear import of PIC [8]. However, PIC’s nuclear translocation relies on several other viral and cellular factors [9].

Functions of the N-terminal domain are the least characterized. Zinc ions binding by this domain was shown to stimulate IN tetramerization and enhance its integrating activity in vitro [10].

The C-terminal domain can bind DNA in a sequence-independent manner. Crosslinking studies suggest that this domain interacts with the subterminal regions of viral DNA [11]. Residues located in the C-terminal domain were shown to be crucial for interaction with viral genomic RNA, and this interaction is important for proper virion morphogenesis [12]. Also, this domain is essential for IN interaction with reverse transcriptase [13,14]. This interaction is crucial for successful reverse transcription [15]. Apart from that, most posttranslational modifications described yet occur in the C-terminal domain (reviewed below).

Despite IN being classically perceived as an enzyme that catalyzes HIV-1 genome integration, the pleiotropic effects of IN mutations on different stages of HIV-1 replication suggest that its functions go beyond the integration process.

1. IN was shown to enhance the initiation and elongation of reverse transcription. Also, direct interaction between IN and RT was proved to be necessary for reverse transcription [15].
2. IN helps to maintain optimal capsid stability in the cytoplasm by promoting interaction between CA and its cellular cofactor CypA. Lack of IN or a mutation C130S in it impairs the interaction between CA and CypA, which leads to defective reverse transcription and a block in replication [16]. This is an indirect mechanism of IN influence on reverse transcription efficiency.
3. The presence of NLS in IN and the fact that IN interacts with cellular proteins involved in macromolecule translocation through the nuclear pore suggest the plausible role of IN in the nuclear import of PIC [17,18,19,20].
4. IN interacts with the HIV-1 RNA genome, and this interaction is necessary for the correct packaging of the genome into viral particles [12]. For the successful interaction, IN must be in a tetramer form. A decreased level of IN, substances that disrupt its multimerization, and mutations that disrupt the RNA—IN interaction lead to the formation of non-infectious virions with eccentric morphology [21].
5. IN interaction with the cellular Ku70 protein, which is a component of DNA-PK, is essential for the successful repair of cellular genome damages resulting from the viral cDNA integration. This process can be disrupted by mutations in integrase that prevent Ku70 binding [22,23].

Given its multiple roles in the HIV life cycle, not surprising is the fact that IN recruits many cellular proteins that may affect not only integration. Interactions between IN and its cellular partners, their structural (where known), and functional aspects constitute the topic of this review. The partners are grouped according to their influence on IN and the different steps of HIV-1 replication.

## 2. Factors Affecting Integrase Stability

**Figure 3:**
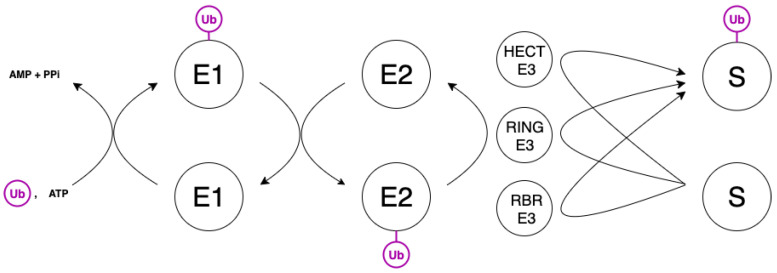
Scheme of the ubiquitination process in mammalian cells. Ubiquitin (Ub) is initially activated by the formation of an acyl phosphate with ATP, which then acylates the cysteine group of the E1 protein. Ub is then transferred from E1 to a cysteine of one of the members of the E2 family, which interact with proteins of the large family of E3 ubiquitin ligases. E3 ligases are usually classified into three main classes (RING, HECT, and RBR) based on conserved structural domains and the molecular mechanism of Ub transfer to the substrate protein. Sites of ubiquitination vary among different substrates. For most proteins, the first Ub moiety is conjugated to an ε-NH2 group of an internal Lys residue.

In mammalian cells, all the proteins are degraded by the ubiquitin-proteasome system (UPS). The degradation serves for protein quality control and homeostasis [24]. Protein ubiquitination consists of several steps and requires three major enzymes (Figure 3). The first one is a ubiquitin-activating enzyme (E1) that binds ATP and then catalyzes a nucleophilic attack of the C-terminal carboxyl group of ubiquitin (Ub) on the α-phosphate of ATP to produce the respective acyl phosphate, which subsequently acylates the cysteine residue of E1 with the formation of a thioester bond between E1 and Ub. Activated Ub is then transferred from E1 to a cysteine residue in a second protein, which is a ubiquitin-conjugating enzyme (E2). Transfer of Ub to substrate proteins typically requires a third enzyme—ubiquitin ligase that catalyzes the formation of an isopeptide bond between a lysine residue of the substrate protein and the C-terminal glycine residue of Ub (E3). Depending on E3, substrate ubiquitination occurs either by direct transfer of Ub from E2 or through the intermediate formation of a thioester bond between Ub and E3 [24,25]. Repeating these steps results in polyubiquitination of the substrate protein.

Ub can be attached to another ubiquitin through any of its seven lysine residues. As a result, various linear or branched Ub chains, which provide different information about the future of a substrate protein, can be formed. Attachments through Lys11 and especially Lys48 are the most well-studied and are believed to serve as a signal for proteasomal degradation [24,26].

Since integrase is important for the successful completion of early replication steps, its premature or increased degradation can impede successful virus replication. Therefore, it is important to determine E3 ligases that recognize and target it for proteasomal degradation. Besides E3 ubiquitin ligases, some other cellular proteins are described as IN-stabilizing proteins that can also influence HIV-1 replication.

### 2.1. TRIM33

RING-type E3 ubiquitin ligase TRIM33 was shown to interact with IN and to be an important factor in determining its degradation [27]. In IN overexpressing cells downregulation of TRIM33 significantly increased IN level and slowed down IN degradation after cycloheximide treatment. TRIM33 knockdown decreased IN ubiquitination level, proving that it acts by stimulating IN ubiquitination. In single-round infection experiments with VSV-G pseudotyped vector carrying luciferase reporter, TRIM33 knockdown promoted infection: the luciferase signal and the number of integrated DNA increased, while the number of 2-LTR circles, a sign of non-productive integration, decreased [27]. This shows that an increase in the IN level does stimulate the early stages of infection. Interestingly, in TRIM33-knockdown cells, both early and late HIV reverse transcripts were also slightly increased. It might be due to the IN effect on the reverse transcription mentioned above. In multiple-round infection experiments with HIV-1 BRU, knockdown of TRIM33 also increased IN level and promoted infection, while TRIM33 overexpression in KD cells impaired infection. All these data suggest that TRIM33 is a major mediator of IN degradation and confirmed the influence of IN concentration on HIV-1 successful replication [27]. It should be also noted that TRIM33 is the only member of the TIF1 subfamily of TRIM proteins that regulates HIV-1 integrase stability [27].

### 2.2. CRL2^VHL^ Complex

One more E3 ubiquitin ligase that is involved in IN degradation is the CRL2^VHL^ complex. It consists of a substrate recognition subunit von Hippel-Lindau protein (VHL), a scaffold protein Cullin-2, adaptor proteins Elongin B and Elongin C, and E3 ubiquitin ligase Rbx1 [28]. VHL was shown to bind IN in presence of von Hippel-Lindau binding protein 1 (VBP1) [29]. VBP1 itself was initially identified as an IN partner by yeast two-hybrid assay and confirmed by coimmunoprecipitation assay in the co-overexpression system [30]. When VBP1, VHL, or Cul2 were downregulated in HeLa cells stably expressing IN-HA, there was less polyubiquitinated IN-HA, and its degradation was slowed down [29]. These observations suggest that CRL2^VHL^ acts as a VBP1-dependent E3 ubiquitin ligase for IN that decreases the IN level and therefore can be considered a negative factor for HIV-1 replication. Surprisingly, when cells were infected with NL4-3Δenv-Luc VSV-G-pseudotyped viral vector, knockdown of CRL2^VHL^ components, VBP1, Elongin B, or Rbx1, resulted in decreased luciferase signaling, and the strongest decrease was found upon VBP1 depletion. A detailed analysis of a replication step affected by the VBP1 knockdown showed that VBP1-targeting siRNA did not significantly affect the amount of either total reverse-transcribed or integrated viral DNA, whereas almost completely inhibited the expression of viral RNA. However, the knockdown of VBP1, Elongin B, and Rbx1 had no effect on luciferase expression from the transfected pNL4-3ΔenvLuc plasmid, indicating that VBP1 is not involved in HIV expression when the integration step is omitted. The authors supposed that VBP1/CRL2^VHL^ regulate viral transcription immediately after the integration stage, but their influence is somehow mediated by interaction with IN. VBP1/VHL-mediated IN degradation is likely to be required for proper transcription of viral genes [29]. However, this hypothesis is to be proved.

### 2.3. hRAD18

Unlike E3 ubiquitin ligases mentioned above, the hRAD18 protein positively regulates the IN stability, although it possesses a RING finger domain [31]. This protein is considered to be involved in post-replication DNA repair [32]. Interaction between IN and hRAD18 proteins was shown by co-IP in co-overexpressing cell lysates [31]. Moreover, the IN co-localization with hRad18 in nuclear structures has been observed in a subpopulation of co-transfected cells. It has been shown that when overexpressed together with IN, hRAD18 increased its level compared to when only IN was overexpressed. This was true for IN with N-terminal Phe (wild type), Arg, and Met, which means that the stabilization was independent of the N-end rule [31]. However, the mechanism of the IN level increase in the presence of hRAD18 and the role of changes in its intracellular distribution has not been determined yet.

### 2.4. LEDGF/p75

One of the best-described IN partners is lens epithelium-derived growth factor/p75 (LEDGF/p75). In a cell, it acts as a transcriptional co-activator of stress-related proteins and protects a cell under different stress conditions [33,34,35]. LEDGF/p75 also plays a role in DNA homologous recombination [36], in the initiation of mixed lineage leukemia (MLL)-mediated leukemia [37], chemoresistance, cell cycle regulation, and survival of MLL-mediated leukemia and solid state tumors [38]. It may also be involved in neurogenesis [39]. The involvement of LEDGF/p75 in HIV-1 infection includes targeting the pre-integration complex to actively transcribed regions, influencing IN localization (these functions will be described in the corresponding sections), and IN stabilization. LEDGF/p75 decreases the polyubiquitination of IN, thus increasing its stability [40]. Interestingly, the stabilizing effect of LEDGF/p75 on IN was observed both in the nucleus and cytoplasm using wild-type LEDGF/p75 with nuclear localization and the mutant variant with cytosolic localization [40].

### 2.5. Ku70

Another protein previously thought to stabilize IN is Ku70, a protein that together with Ku80, forms a Ku heterodimer which is involved in DNA repair by the NHEJ pathway. Ku70 was shown to interact with IN in the yeast two-hybrid system [41], by co-IP in the co-expression system, and when C8166 CD4+ T cells were infected with HxBru-IN-HA [42]. When overexpressed, Ku70 stabilized IN, and vice versa when its expression was suppressed, the level of IN decreased [42]. This could be explained by the direct interaction of two proteins or by other mechanisms independent of the interaction. One such mechanism may rely on the deubiquitinating ability of Ku70 in the cell [43]. Using an IN mutant with impaired Ku70 binding, it was shown that both the mutated protein and wild-type IN have a similar half-life and degradation kinetics when overexpressed in cells with different intracellular Ku70 levels [23]. This observation proves that direct IN-Ku70 interaction is dispensable for IN stabilization. Since Ku70 has been later shown to be an important participant in post-integrational DNA repair, it will be described in detail in the corresponding section.

## 3. Factors Affecting Integrase Acetylation

Histone acetyltransferases (HATs) and histone deacetylases (HDACs) manage the acetylation of histones, serving as important epigenetic regulators. Many of them also have non-histone protein substrates. Acetylation and deacetylation of non-histone proteins play an important role in a variety of cellular processes because they can affect protein functions (reviewed in [44]). A possibility of non-enzymatic protein acetylation was also suggested [44]. Here we describe HATs and HDACs that have been found to interact with HIV-1 IN.

### 3.1. p300

Histone acetyltransferase p300 directly binds to and acetylates HIV-1 integrase at its C-terminal domain residues K264, K266, and K273 [45,46]. Acetylation by p300 enhanced the DNA binding activity of IN and the efficiency of strand transfer reaction in vitro, while not affecting 3′-processing [45]. In line with in vitro observations, the HIV-1 BRU vector carrying K(264,266,273)R mutant IN showed a very low replication level in CEM cells and was almost undetectable in PBLs. A similar result was found for HIV-1 BRU without mutations but in the presence of p300-specific inhibitors. The authors demonstrated that this replication decrease was due to impaired integration [45]. However, these results were left in doubt in [46] where HIV-based vector coding for IN carrying the same triple mutation was replication competent. It should be noted that in [45], HIV-1 BRU contained IN with a FLAG-tag located at the end of the C-terminal IN domain just near the target site for acetylation, whereas in [46], IN was not tagged. This fact can explain the conflicting results of these works since although the wild-type tagged virus is viable, the combination of this FLAG-tag with the K(264,266,273)R substitution can significantly reduce the efficiency of integration. Nevertheless, a certain effect of K(264,266,273)R substitutions on IN activity obviously exists since even in the case of untagged IN, the presence of this triple substitution led to a 1.7-fold decrease in the amount of integrated DNA and a 4-fold increase in 2-LTR circles signaling about less effective integration than for wild-type IN [46]. The negative influence of K(264,266,273)R substitutions on integration were also confirmed in [47].

### 3.2. GCN5

Another HAT, GCN5, has also been shown to interact with HIV-1 IN and to acetylate it at K258, K264, K266, and K273 [48]. The acetylation promoted both 3′-processing and strand transfer reactions in vitro. In vivo experiments with VSV-G pseudotyped NL4.3 virus expressing the luciferase reporter gene confirmed the importance of IN acetylation by GCN5 for integration reaction and its influence on HIV-1 infection kinetics. Of note, viral vectors used to show the latter contained untagged IN. Viruses encoding the K(258,264,266,273)R and K(264,266,273)R IN mutants showed a similar five-fold decrease in infectivity compared to the wild type, which arose due to the reduced integration. The comparative analysis of the replication efficiency of IN triple and quadruple mutant viruses convincingly showed that acylation of K264, K266, and K273 by both GCN5 and p300 is necessary for efficient integration of the virus, while specific modification of K258 by GCN5 does not affect it [48].

Directly opposite results were obtained in [49]. The role of substitution of K258, K264, K266, and K273 was investigated using pNL4.3R-E-luciferase reporter viral vector. The luciferase level was significantly reduced by both K258R (3-fold decrease) or K(258,264,266,273)R (50-fold decrease) mutations, while single K264R, K266R, or K273R mutations had no effect. Quantification of total reverse transcription products and 2-LTR circles unexpectedly demonstrated a 3-fold reduction in total reverse transcription products and a comparable level of 2-LTR circles for both K258R and quadruple mutants. This result allowed the authors to consider that all the K to R substitutions had no considerable effect on HIV integration, however, it could not explain the 50-fold decrease in expression of a luciferase reporter gene in the case of the quadruple mutant. Further investigation showed that the observed effect of the K(258,264,266,273)R mutant could be explained by its influence on early transcriptional events after integration, as there was an average 80-fold decrease in the level of transcripts produced from proviruses integrated by the mutant IN against wild type IN. The proviral DNA of the virus with the mutant IN was found to be less associated with H3K27ac (the marker of active transcription) than in the case of wt provirus 2 days post-infection. The effect of the mutant IN on chromatin modifications present at the viral LTR was transient, and by five days post-infection, the levels of the LTR associated with H3K27ac were comparable for both viruses. Interestingly, the IN quadruple mutation had no significant effect on the association of histone H3 with proviral DNA, demonstrating that there was no defect in the loading of histones onto the viral DNA. S. Winans et al. also demonstrated the lower binding of IN quadruple mutant with viral DNA and suggested the possible mechanism for the observed difference in the association of proviral DNA with H3K27ac. IN is no longer retained on the proviral DNA and is unable to recruit host factors that would normally play a role in activating proviral transcription through chromatin modification. Thus, the repressive chromatin state present on the unintegrated viral DNA may remain intact for longer times, causing low proviral transcription even after integration [49].

It is worth adding that an additional study of the K258R mutation showed that it redirects integration to centromeric alpha satellite repeat sequences and thus leads to latency [50]. However, experiments with mutants cannot distinguish between effects due to lack of acetylation and some other possible reasons.

### 3.3. HDAC1

Histone deacetylase HDAC1 has been identified as HIV-1 IN partner in co-immunoprecipitation and pull-down experiments [51]. IN was shown to be acetylated by HDAC1 [52]. HDAC1 knockdown in HeLa cells prior to the transduction by HIV-1 NL4–3 was shown to interfere with the early preintegration step of the HIV-1 replication cycle, which possibly involves reverse transcription. Analysis of the HDAC1 influence on the expression of proviral DNA showed some surprising results since HDAC1 overexpression decreased HIV-1 gene expression, while HDAC1 knockdown had no effect. Transient knockdown of HDAC1 in HIV-1 producer cells differently impacts replication in infected cells, namely, it had no effect on virus infectivity in CD4+ HeLa and LuSIV cells, whereas a slight, albeit significant, increase in infectivity in both Jurkat and human primary CD4+ T cells was observed [51].

However, another group had the opposite result since the knockdown of HDAC1 in HeLa cells increased the amount of integrated DNA and reduced the amount of 2-LTR circles in infected cells. Treatment of cells with a potent inhibitor of class I and II mammalian histone deacetylases, TSA, or a specific inhibitor of HDAC1 catalytic activity, MS-275, also increased the level of integrated DNA [52].

The requirement of virion-associated HDAC1 to HIV-1 replication was also analyzed in [53] using two approaches: RNA-interference mediated knock-down of HDAC1 in producer cells and incorporation of an inactive mutant of HDAC1, which decreased the virion-associated histone deacetylase activity. In both cases, decreased virion-associated HDAC1 activity led to a reduction in the infectivity of these virions in target 293T cells specifically at the early reverse transcription stage, while the entry of the virions was unaffected.

The observed inconsistency in the results on the effect of HDAC1 on HIV-1 replication can be explained both by different experimental conditions in different studies and by the fact that in the cell, HDAC1 interacts with a large number of proteins and is part of various complexes, the components of which can also interact with IN. Thus, HDAC1 is associated Sin3a complex that also contains SAP18 (Sin3a-associated protein, 18 kDa) [54], which was shown to interact with IN [53]. Moreover, a well-known IN partner INI1 [55] (see below for INI1 influence on HIV replication) is also associated with components of the Sin3a-HDAC1 complex in vivo [53]. Given that HIV-1 IN and INI1 bind SAP18 and selectively recruit components of the Sin3a-HDAC1 complex into HIV-1 virions, it is difficult to unambiguously understand which factor affects the infectivity of these virions in target cells.

### 3.4. KAP1, Link with HDAC1

Another IN partner involved in the regulation of its deacetylation together with HDAC1 is the cellular protein KAP1 (TRIM28) [52]. KAP1 is an epigenetic corepressor that is recruited to its targets by Kruppel-associated box domain zinc finger proteins (KRAB-ZFPs). KAP1 then mediates transcription repression by recruiting repressive histone modifying factors such as the histone methyltransferase SETDB1, the histone deacetylase NuRD complex, and heterochromatin protein 1 (HP1). Together they suppress transposable elements [56]. KAP1 is known to suppress HIV-1 gene expression and to be involved in the establishment and maintenance of the latent state of the virus [57,58]. It also interacts with the Tat protein and promotes its proteasomal degradation, thereby reducing the efficiency of viral transcription [58].

KAP1 was shown to interact with IN, and this interaction is highly favored by IN acetylation [52]. Knockdown of KAP1 resulted in an increased level of integrated DNA during infection of HeLa cells with the pNL4.3-Luc vector, and overexpression of KAP1 inhibited HIV-1 infection due to a decrease in the amount of integrated DNA. Of note, any change in the KAP1 level did not influence the HIV integration when the pNL4.3-Luc-3mut vector carrying IN mutated in the lysines targeted for acetylation (K(264,266,273)R) was used. Further experiments have demonstrated that the KAP1 impaired HIV-1 replication by recruiting HDAC1 deacetylase to IN since a higher level of HDAC1 associated with IN was detected in cells overexpressing KAP1 as compared to control cells [52]. These data clearly demonstrate that KAP1 induces IN deacetylation by favoring HDAC1 binding to IN.

### 3.5. HDAC10

In contrast to HDAC1, the effect of HDAC10 on HIV replication is in no way related to IN deacetylation. HDAC10 is downregulated during HIV-1 infection due to the activity of virus-associated envelope glycoprotein [59]. Knockdown of HDAC10 in CD4+ T cells with specific shRNAs was shown to benefit HIV-1 infection, specifically facilitating an integration step [60]. HDAC10 was found to interact with IN, and its binding site is located in the region of residues from 55 to 165 in the catalytic domain of IN. Importantly, HDAC10 did not significantly change the lysine acetylation state of IN but weakened its interaction with LEDGF/p75. Since this interaction is important for IN stability [40] and efficient integration into actively transcribed genome regions (see below), its disruption led to impaired viral integration, which explains the negative effect of HDAC10.

## 4. Factors Affecting Integrase Phosphorylation

Protein phosphorylation is a reversible post-translational modification of proteins performed by protein kinases that modify amino acids by adding a covalently bound phosphate group. The amino acids most commonly phosphorylated in eukaryotes are serine, threonine, and tyrosine, and these phosphorylations play important and well-characterized roles in signaling pathways and metabolism. However, some other amino acids, including His, Arg, Lys, Asp, Glu, and Cys, can also be phosphorylated [61]. Phosphorylation rapidly and reversibly regulates cellular signaling functions, impinging on catalytic activity, protein:protein/DNA/RNA interactions, protein localization, and stability [62]. To date, two protein kinases, JNK, and GCN2 are shown to phosphorylate HIV-1 IN.

### 4.1. JNK and Pin1

Manganaro et al. have discovered IN phosphorylation by c-Jun N-terminal kinase (JNK) when studying reasons for inefficient HIV infection in quiescent human peripheral blood T lymphocytes (PBLs) [63]. They have observed several mitogen-activated protein kinases (MAPKs) are not expressed or active in resting CD4+ T cells and become activated in response to T cell stimulation. Inhibition of one of them, JNK, significantly impaired viral infection in PHA-IL2-activated T cells. JNK inhibition led to an increase in early and late reverse transcripts but a decrease in integrated HIV-1 DNA and 2-LTR circles during infection of activated PBLs. These observations suggest that JNK activity is necessary for efficient integration [63].

JNK was shown to interact with integrase in vitro and in vivo and to phosphorylate it at highly conserved Ser57 located in the IN core domain [63]. Phosphorylated IN becomes a substrate for the cellular peptidyl prolyl-isomerase enzyme Pin1, which catalyzes a conformational modification of integrase. S57A mutation did not affect strand transfer activity in vitro, however, decrease Pin1 binding to IN. The same effect can be observed during the treatment of the cells with a JNK inhibitor which prevents IN phosphorylation [63]. Pin1 is the only known peptidyl-prolyl cis/trans isomerase that specifically binds phosphorylated S/T-P motifs and catalyzes the isomerization of the peptidyl-prolyl bond. The isomerization can affect a target protein’s folding, localization, activity, stability, etc. [64]. In the case of IN, this isomerization takes place too, which significantly affects IN stability, since the S57A mutant was less stable than wild-type IN when they were overexpressed in HeLa cells. Pin1 inhibition led to decreased stability of wild-type IN, but not of the S57A mutant, suggesting the importance of S57 phosphorylation for IN stability maintenance and the role of Pin1 in this process [63].

During infection of primary CD4+ T cells or SupT1 T cells with HIV-1 BRU, replication efficiency of the virus carrying IN S57A mutant was seriously decreased due to decreased integration, while reverse transcription and virion production were not affected. Cell treatment with the proteasome inhibitor MG132 eliminated the difference in the level of overexpressed wt and S57A mutant IN. While having no effect on wt HIV-1 BRU vector integration, MG132 partially restored the level of integrated DNA for the S57A mutant HIV-1 BRU. This data reveals that IN S57 phosphorylation by JNK and subsequent isomerization by Pin1 promote the integration step by increasing IN stability. Authors suggest that low expression of JNK in primary resting CD4+ T cells from peripheral blood restricts efficient HIV-1 infection [63].

The importance of HIV-1 integrase interaction with Pin1 was also confirmed in [50]. Direct infection in resting CD4+ T cells is inefficient in vitro, but HIV-1 integration and latency occur in these cells in vivo and in some specific conditions, including treatment of cellular culture with chemokines that bind CCR7, CXCR3, or CCR6 chemokine receptors that are expressed in resting CD4+ T cells. Saleh et al. used resting CD4+ T cells treated with CCR7 ligand CCL19 to study latency establishment. After infection with HIV NL4-3, they observed IN binding to Pin1, which was impaired by a JNK inhibitor. The same effect was observed upon cell activation with PHA-IL2. Both in CCL19 and PHA-IL2 treated cells, Pin1 knockdown led to a reduction in integrated DNA [65].

### 4.2. GCN2

In addition to JNK, IN has also been shown to be phosphorylated in vitro and in vivo by GCN2 (eIF2AK4) [66]. GCN2 (general control non-derepressible-2) is a serine/threonine kinase that is responsible for sensing amino acid deprivation to induce Integrated Stress Response. Upon sensing amino acid starvation, GCN2 changes its conformation, allowing autophosphorylation. The autophosphorylated form of GCN2 is its active form that is potent to trigger further Integrated Stress Response [67].

GCN2 was shown to be activated upon HIV-1Laì infection of HeLa P4 cells. Authors observed an MOI-dependent decrease in general translation level upon infection. GCN2 knockdown in infected cells increased viral replication [68].

GCN2 was shown to phosphorylate IN at S24 and S255, and the latter was identified as a major phosphorylation site in vitro [66]. Interestingly, S255 in CTD was not phosphorylated in vitro in the absence of CCD. Serine-to-alanine and serine-to-aspartic acid mutations at positions 24 and 255 did not alter the level of total DNA, IN stability, or localization, they also did not change the selectivity for specific genomic features or histone modifications at the integration site. At the same time, viral vectors carrying IN S(24,255)A and S255A mutants showed increased infectivity and level of integrated DNA during single-round infection in HEK293T cells, while the IN S(24)A substitution had no effect. Single-round infection of wild-type viral vector was enhanced in GCN2−/− knockout MEF cells. This effect was weakened for IN S(24,255)D and S255D viruses, which can be explained by aspartic acid mimicking the phosphorylated state. The authors have observed a decreased 3′-processing rate in S(24,255)D and S255D mutants [66]. These results suggest that, in contrast to JNK, IN phosphorylation by GCN2 has a negative effect on HIV replication, possibly by weakening integrase catalytic activity.

## 5. Factors Affecting Integrase Sumoylation

SUMOylation is a reversible post-translational modification of ε-amino groups of Lys residues in proteins by covalent attachment of a small ubiquitin-related modifier (SUMO) proteins. SUMO conjugation necessitates an enzymatic cascade resembling that of ubiquitination and involves a single activating E1 enzyme, a single conjugating E2 enzyme (Ubc9, which will be discussed later), and one of a limited set of ligating E3 enzymes [69]. Sumoylation can change the stability and activity of sumoylated proteins, alter their intracellular localization, or affect protein-protein interactions. Additionally, a conservative surface in SUMO proteins can be recognized by SUMO interacting motifs (SIMs) in so-called SUMO readers [69,70]. By simultaneous modification of sets of functionally related proteins, SUMO affects many cellular processes, such as gene expression, cell cycle progression, RNA processing, etc. [69].

IN has been demonstrated to interact with SUMO-conjugating enzyme ubc9 and to be SUMOylated in in vitro SUMOylation assay and in in vivo overexpression systems by SUMO1, SUMO2, and SUMO3 [71,72]. As suggested by sequence analysis, the positions of SUMOylated lysines might be 46, 136, and 244 [72]. The classical SUMOylation motif has a consensus ψKxE, where ψ is a bulky hydrophobic amino acid and x is any amino acid [73]. SUMOylation of the lysines can be experimentally assessed using mutant proteins with replaced lysines or glutamic acids in +2 positions. Triple K(46,136,244)R and E(48,138,246)Q mutations led to a significant decrease in the IN SUMOylation in the co-overexpression system. However, all single and double mutants tested showed a SUMOylation level by any of the three SUMO proteins similar to that of the wild type [72].

The function of SUMOylation of IN is not completely clear. Integrase life span was the same for the wild type and the triple lysine-to-arginine mutant, suggesting that SUMOylation does not influence the rate of its proteasomal degradation [72]. When FLAG-IN is overexpressed in HeLa, its localization is mostly nuclear and stays the same for both the wt protein and the triple lysine-to-arginine mutant that is less SUMOylated [72]. That is also true for GFP-IN which was demonstrated in [74]. However, in another study, eGFP-IN overexpressed in HEK293T was distributed evenly and diffusely, and its localization changes to both diffuse and intranuclear punctate (implying formation of IN aggregates) when it is coexpressed with SUMO1 or SUMO2 + SUMO-conjugating enzyme Ubc9 [71]. Different initial localization of IN might be explained by different cell lines or tags. There is no evidence that SUMOylation influence other post-translational IN modifications [72].

The effect of IN SUMOylation on HIV replication was studied in several works, and the results obtained are rather controversial. Thus, using an HIV-1-derived lentiviral vector system with the EGFP reporter gene, it was shown that downregulation of endogenous SUMO1/SUMO2 and Ubc9 increases EGFP expression and HIV-1 integration, while superexpression of SUMO1/SUMO2 and Ubc9 inhibit them [71]. At the same time, a decrease in infectivity was demonstrated for viral vectors carrying the triple K(46,136,244)R mutation that impairs IN SUMOylation. The same triple mutation in IN did not affect its catalytic activity [72]. A decrease in infectivity is also observed in single-round viruses carrying IN with the E(48,138,246)Q mutation, which also disrupts SUMOylation. These results indicate that the impairment of IN SUMOylation causes a decrease in infectivity [72]. It can also be added that the downregulation of Ubc9 in HEK293T was shown to decrease HIV-1 infectivity, probably by influencing virion morphogenesis [75]. At the same time, overexpression of Ubc9 in HEK293T-producing cells had no effect on the virus infectivity in another study, while overexpression of SUMO1 led to the production of less infectious virions [76]. All this information suggests that SUMOylation pathway components must have multiple effects on the HIV-1 life cycle, and the interplay between them, as well as the role of the IN SUMOylation, is to be explained.

As it is mentioned above, SUMOylated proteins can often establish non-covalent protein-protein interactions with their binding partners. The vast majority of these interactions involve a conserved surface in the SUMO protein and a SUMO interacting motif (SIM), a short stretch of hydrophobic amino acids, and an acidic region, in the interactor protein called SUMO reader. Apart from being covalently SUMOyled, IN was proposed to act as a SUMO reader [74]. Bioinformatics analysis of the IN amino acid sequence revealed three putative SIMs: SIM1 (72VILV75), SIM2 (200IVDI203), and SIM3 (257IKVV260). Mutations in all three SIM domains (triple mutant) as well as in SIM2 or SIM3, but not in SIM1, impaired IN binding to SUMO3 protein. These data demonstrated that HIV-1 IN is capable of interacting directly with SUMO3, and at least two SIMs in IN (SIM2 and SIM3) are involved in this noncovalent binding. The study of the potential effect of IN/SUMO3 binding on its own SUMOylation demonstrated that the SUMOylation level of the IN triple mutant was approximately four times greater than that of the wt IN, suggesting that IN-SUMO3 non-covalent interaction through SIMs humpers IN SUMOylation [74]. Interestingly, mutations in all SIMs of IN significantly stimulated its binding to the SUMO E2-conjugating enzyme Ubc9, which is indispensable for the SUMOylation of all proteins. Accordingly, the increased binding capacity of the IN triple mutant to Ubc9 resulted in its increased SUMOylation level [74].

It was also shown that mutations in SIM2 or SIM3 lead to decreased IN binding to LEDGF and increased binding to Ku70. Apart from that, further investigation revealed that triple mutant has more cytoplasmic localization than GFP-IN wt. Consistently, the authors showed that the mutations in SIM2 or SIM3 humper early steps of infection. These results suggest that the original amino acid composition of these motifs is important for IN functioning [74]. However, the fact that these effects are mediated by SUMO noncovalent binding is to be proved.

To conclude this section, it should be noted that interplays between viruses and the host SUMOylation system are very complex. Viruses can manipulate the SUMO pathway to promote viral replication and pathogenesis, and in particular, HIV-1 can impair cellular SUMOylation by targeting the host’s SUMO E1-activating enzyme [77].

## 6. Factors Affecting Nuclear Import of Integrase

In a non-dividing state of the cell, its nucleus is surrounded by a membrane, and transport of macromolecules is only possible through a nuclear pore complex (NPC) built from proteins named nucleoporins. NPC is freely permeable to small molecules, but imposes stringent selectivity on the passage of proteins and RNA, tightly regulating their traffic. Transport of almost all macromolecules in and out of nuclei is an active process that requires the assistance of soluble proteins called nuclear transport factors (NTFs). NTFs recognize cargoes that have to be transported in or out of the nucleus by specific amino acid signals called nuclear localization sequences (NLS) or nuclear export sequences (NES), respectively. NTFs bind the cargoes and assist their transport through the nucleoporin network to pass the NPC channel [78].

As a member of the lentivirus genus, HIV-1 is capable of infecting non-dividing cells by transporting PIC to their nuclei [79]. For a long time IN was believed to be a key player in this process.

The ability of IN to penetrate the cell nucleus has been proved in multiple experiments with IN fused to different labels, GST, BSA, HA, and Cy3, the nuclear localization of which can be easily detected [19,80,81]. Nuclear import of IN was inhibited completely by WGA [81,82], a lectin that binds nucleoporins, thus inhibiting specific transport processes without affecting diffusion [83]. In addition, nuclear accumulation of IN was not observed when the nuclear transport reaction was carried out at 4 °C [81,82]. These observations suggest that IN is actively transported into nuclei through the NPC, and also indicate the presence of NLS within IN for the binding to some NTPs.

Many works were aimed at searching for NLS within IN and determining amino acid residues important for its nuclear import. Thus, bipartite NLS 186–189 & 211–219 [19,84], regions 161–173 [17,18], and 251–270 [20] were suggested as putative NLS. However, their role in binding NTFs and nuclear translocation of IN was questioned in other works [85,86,87]. Thus, IN is able to actively penetrate the cell nucleus through NLC, but the exact mechanism of this transport and its role in HIV-1 infectivity remains to be elucidated. Now we will focus on probable factors involved in IN nuclear import.

### 6.1. Importin α

Nuclear import and export of most proteins are mediated by members of a large family of NTFs known collectively as karyopherins. Karyopherins include importins (facilitate nuclear import) and exportins (facilitate nuclear export), and all karyopherins interact directly with their cargoes, although some also use adapter proteins. The best-characterized adapter protein is karyopherin α (also called importin α) [88], which has several isoforms [89].

IN was shown to interact with importin α (Imp α) in vitro and in vivo [17,19,90,91]. ALPHAScreen assay revealed that IN interacts with both Imp α and Imp β proteins alone and with the Imp α/β heterodimer, however, the affinity for Imp α and Imp α/β is significantly higher than for Imp β [90]. The latter shows that IN interacts with the Imp α subunit of the Imp α/β heterodimer, and therefore it might be imported into the nucleus via the Imp α/β-dependent pathway. This assumption was confirmed by a significant decrease in the IN nuclear accumulation when antibodies to both Imp α and Imp β were added. Participation of Imp α in the IN nuclear import was confirmed in [91] but refuted in [80,92].

This discrepancy might be explained by the usage of different importin variants. Humans have 7 isoforms of importin α. Despite the high similarity in amino acid sequence and 3D structure, they display remarkable substrate specificity in vivo [89]. In [81,92], Imp α1 (Rch1) was shown to not be involved in IN nuclear import, and the results were extrapolated to the whole group of Imp α proteins. At the same time, a more detailed study of the Imp α family proteins showed that, when Imp α1, Imp α3, Imp α5, and Imp α7 were knocked down in HeLa cells, CD4+ C8166 T cells, and primary macrophages, Imp α7 depletion had no effect on HIV-1 replication, depletion of Imp α1 and Imp α5 had a slight effect, and only Impα3 knockdown significantly impaired HIV infection. Imp α3 depletion resulted in a reduced level of 2-LTR circles and integrated DNA while not affecting reverse transcription, which suggests its role in nuclear import. IN was clearly shown to interact with Imp α3 both in a 293T cell expression system and in HIV-infected CD4+ C8166 T cells via its region from 250 to 270 amino acids, which was suggested as NLS [20].

It can be assumed therefore that for the nuclear transport of IN, its interaction with Imp α3 is important; other proteins of this family are unlikely to be involved in this process.

### 6.2. Importin 7

Apart from Imp α, an importin 7 (Imp 7) is another importin that is well-studied in terms of its influence on HIV-1 replication and IN nuclear import. It is an NTF that can act autonomously or as a heterodimer with importin β [93].

IN was shown to interact with Imp 7 [92,94,95], and this interaction is rather specific, since INs from HIV-2, SIVmac, and BIV only weakly interacted with Imp 7, and no detectable interaction was observed for EIAV IN. Two regions within IN, 235WKGPAKLLWKG245 and 262RRKAK266, were shown to be required for efficient interaction between IN and Imp7 [92]. VSV-G pseudotyped HIV-1 viral vector carrying IN mutant unable to bind Imp 7 was less infectious, with both reverse transcription and nuclear import being affected [92]. A decrease in HIV-1 replication was also observed when Imp 7 was depleted [94,95]. Taken together, these data highlight the importance of Imp 7 for HIV-1 replication and suggest the involvement of its interaction with IN.

### 6.3. Transportin-SR2 (TNPO3)

Transportin-SR2 (TRN-SR2, TNPO3) is a protein from the Imp β family. Its role in the nuclear import of the HIV-1 genome is extensively studied and discussed in detail in [96]. Here we will focus on its interaction with HIV-1 IN.

TRN-SR2 was identified as an IN partner by yeast two-hybrid screening and confirmed by pull-down assay [97,98] and co-IP in virus-infected cells [91]. Amino acids 262RRK264 and 266KIIR269 within IN were determined as key residues involved in TRN-SR2 binding [99,100]. A virus carrying mutant IN with decreased affinity for TRN-SR2 completely retained wild-type reverse transcription activity, while viral DNA nuclear import and integration were impaired [101]. Importantly, disruption of nuclear import was shown both by qPCR and by analysis of eGFP-labeled IN localization using fluorescence microscopy.

The participation of TRN-SR2 in the IN nuclear transport was observed in [91], where peptides that have been shown to promote dissociation of the IN-TRN-SR2 complex inhibited the nuclear import of IN in infected cells. Knockdown of TRN-SR2 disrupted HIV-1 replication in HeLaP4 cells and primary macrophages, and qPCR analysis indicated blocking of PIC nuclear import, more precisely, the stage between late reverse transcription and the nuclear entry step. Moreover, analysis of the localization of PIC with eGFP-labeled IN by confocal microscopy showed that, compared with control cell lines, the vast majority of cells treated with siRNA to TRN-SR2 practically did not contain PIC in the nucleus [97]. Experiments with TRN-SR2 knockout cells also confirmed that it is directly involved in the nuclear import of HIV-1 IN [102]. These observations suggest that interaction between IN and TRN-SR2 may be involved in HIV-1 genome nuclear import.

Importantly, there is an alternative hypothesis, according to which TRN-SR2 exerts its role in HIV-1 nuclear import through direct interaction with the viral capsid protein (CA) and not with IN [98,103]. This hypothesis is based on the fact that certain CA mutations, such as N74D, reduce the dependence of HIV-1 replication on TRN-SR2 in single-round infection assays. However, in multiple-round infection experiments, the virus carrying the N74D CA mutation remained sensitive to TRN-SR2 depletion [104]. The authors consider that these capsid mutations affect virus uncoating and that this represents a rate-limiting step prior to HIV nuclear import.

Overall, TRN-SR2 is an important factor in HIV-1 genome nuclear import, but the role of its interactions with IN in this process is debatable.

### 6.4. NUP153

Apart from recruiting NTFs, IN might interact directly with NPC. IN was shown to interact with nucleoporin 153 (NUP153), which is a component of NPC. IN directly binds to the FxFG-rich C-terminal domain of NUP153, a sequence thought to facilitate nuclear transport of diverse nuclear import complexes by binding to conserved motifs present on importin proteins [82]. NTF2, a protein that interacts with NUP153, was shown to decrease the efficiency of IN binding to NUP153 in vitro, probably by competing with IN and inhibiting IN nuclear import in semipermeabilized cell assay. Overexpression of the NUP153 C-terminal domain was shown to inhibit viral replication, probably due to the binding of exogenous NUP153 to IN in the cytoplasm where it is localized, which prevents IN from interacting with NPC [82]. An interesting hypothesis was proposed regarding the role of NUP153 in the nuclear import of PIC. Depending on the transport status of the pore, the localization of NUP153 can vary from the nuclear to the cytoplasmic surface of the NPC, and increased transport activity through the pore promotes NUP153 exposure to the cytoplasm. Then, the stimulation of nuclear import of IN by Imp α proteins described above can occur not due to their direct interaction with IN, but due to the general stimulation of transport activity caused by them, which leads to an increase in the exposure of NUP153C on the cytoplasmic surface of NPC and facilitates its interaction with IN for nuclear import [82].

Of note, the potential role of NUP153 in the nuclear import of HIV-1 does not exclude the possible role of other nucleoporins in this process. Transport of a large complex like the PIC may require multiple contacts between PIC-associated factors and NPC constituents. In addition to NUP153, the siRNA screening study identified several nucleoporins that are essential host factors for HIV-1 replication [105].

**Figure 4:**
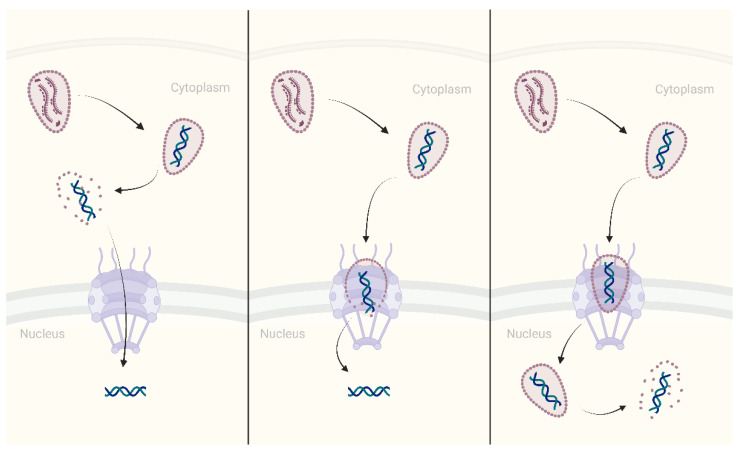
Models of HIV-1 capsid uncoating. Capsid uncoating starts almost immediately after HIV-1 core enters the cytoplasm following virus–cell membrane fusion (left panel). Capsid uncoating initiates when the virus core is docked on the NPC (central panel). The intact or nearly intact HIV-1 core penetrates through the NPC and uncoating occurs near sites of integration in the nucleus (right panel).

Concluding this section, it should be noted that to date, three major models have been proposed that describe the nuclear transport of HIV-1 (Figure 4): 1. capsid uncoating starts almost immediately after HIV-1 core enters the cytoplasm following virus-cell membrane fusion; 2. capsid uncoating initiates when the virus core is docked on the NPC; 3. the intact or nearly intact HIV-1 core penetrates through the NPC and uncoating occurs near sites of integration in the nucleus [106,107]. Direct involvement of IN in the nuclear import of PIC is only possible in the first and, partially, in the second model, whereas the third model practically excludes the participation of IN in nuclear transport. The third model is supported by several recent works [108,109,110,111] including direct visualization using cryo-electron tomography, showing that intact HIV-1 capsid passes through the NPC and uncoating occurs inside the nucleus [112,113]. However, the first two models are also supported by data from various studies from different groups [114,115,116]. In particular, it was shown using live-cell fluorescent imaging that HIV-1 uncoating leading to infection is a cytoplasmic process that occurs ∼30 min postfusion [114]. Thus, a consensus view of HIV-1 uncoating has not yet been achieved. It is for this reason that we have chosen to describe here the cellular factors involved in the nuclear import of HIV-1 IN.

## 7. Factors Affecting Integration Site Selection

After passing through the nuclear pore, PIC is localized in the nuclear periphery, which is a complex and inhomogeneous structure. It is known that generally, HIV-1 integrates its genetic material into cell genome fragments undergoing active transcription [117]. An analysis of approximately 1 million integration sites in HEK293T cells transduced with a single-round HIV-1 vector revealed that nearly 75% of them were located in active genome regions [118].

In 2019, it was shown that the HIV-1 genome might be integrated into enhancer-rich parts of the genome [119]. Just later, it was found that integration might take place in speckle-associated regions [120] (speckles are nuclear bodies formed by splicing factors and pre-mRNA [121]). These data have been confirmed in a very recent study [122]. The authors postulate that nuclear speckle-associated chromatin and chromatin adjacent to speckles are efficient prognostic markers of integration. Moreover, they disprove that the HIV-1 genome generally integrates into introns [118] and hypothesize that genes without introns adjacent to nuclear speckles are more favorable than those containing an intron. These conclusions require additional testing, therefore, as for now, it is impossible to postulate that nuclear speckles are responsible for integrase targeting.

The fact that integrase per se is not specific to any specific parts of cellular DNA [5,6] suggests that there are its cellular partners, which, for example, are able to recognize some epigenetic marks and thereby promote integration into certain sites.

### 7.1. LEDGF/p75

The selection of the viral integration site is conditioned by numerous cellular factors. Among them, the most important and the best studied is LEDGF/p75 (lens epithelium-derived growth factor). The peculiarities of its interaction with IN and the structure of their complex are summarized in various reviews [123,124,125]. LEDGF/p75 includes a C-terminus IBD domain (IN-binding domain) and a conservative N-terminus PWWP domain (Pro-Trp-Trp-Pro) [126]. Using co-immunoprecipitation, LEDGF/p75 was found to interact with IN in the cell [127] and later it proved to play a key role in HIV-1 replication [128]. LEDGF/p75 binds IN through the IBD and due to the interaction of the PWWP domain with the H3 histone trimethylated on Lys36 (H3K36me is a marker of chromatin undergoing active transcription) directs IN to the actively transcribed regions [129]. When LEDGF/p75 levels are decreased by a knockdown or a knockout, the viral infectivity decreases [130], and the integration sites translocate to low-activity regions of the genome [131]. Of note, knockout of the gene encoding LEDGF/p75, PSIP1 [132], but not its knockdown with the help of siRNA, had a much stronger effect on integration [129,130,133]. Probably, a residual level of LEDGF/p75 may be sufficient for recruiting IN to actively transcribed regions of the genome. To preserve the cellular functions of LEDGF/p75, a cell line with mutant LEDGF/p75 (D366N) unable to bind IN was prepared using CRISPR/Cas9. As with LEDGF/p75 depleted cells, D366N cells did not support HIV replication, in part due to decreased integration efficiency. In addition, the remaining integrated provirus was more silent [134].

The interaction of IN and LEDGF/p75 has been widely studied. In 2007, IN residues W131, I161, R166, Q168, and E170 were found to be responsible for the interaction with LEDGF/p75 in vitro [135]. Of note, mutations of these residues did not decrease the enzymatic activity of IN. In vivo experiments showed that besides E170, IN residues H171 and L172, located in the ^170^EHLK^173^ loop region between helices α4 and α5, are also responsible for LEDGF/p75 binding [136]. These mutations of IN significantly impaired its interaction with LEDGF/p75 but had no significant effect on the IN’s ability to bind chromatin. Therefore, there may be alternative mechanisms for IN binding to host chromatin.

Besides influencing integration site selection, LEDGF/p75 may also contribute to the stabilization of the functional multimer state of IN. IN is known to function as a tetramer [137,138] and the dimer-dimer interface is important for tetramerization [139], concerted integration in vitro, and HIV-1 infectivity [127]. LEDGF/p75 is able to bind both the IN dimer [140] and tetramer [127], and it also favors the multimerization of wild-type IN and its mutant variants with decreased multimerization capacity: H12N, E11K, Y15A, D25A, K186E, K188D, and I191E [139].

Interestingly, integration site selection may be influenced by other factors besides IN partners. Such factors include the CPSF6 cellular protein (cleavage and polyadenylation specific factor 6). CPSF6 is a component of a large protein complex of pre-mRNA splicing factors and transports mRNA between the nucleus and the cytoplasm [141]. CPSF6 is not an IN partner, but it was shown to interact with the capsid protein (CA) before PIC translocation into the nucleus [142]. It has also been shown that its knockout suppressed integration into actively transcribed chromatin regions [143]. The knockout of CPSF6 together with LEDGF/p75 decreased integration, while the knockout of CPSF6 predominantly influenced the choice of integration site [143]. Moreover, regardless of the presence of LEDGF/p75, PIC is mainly localized in euchromatin, which may indicate the involvement of the CA-bound CPSF6 in the recruitment of PIC to certain regions of the genome [144].

### 7.2. HRP2

HRP2 (hepatoma-derived growth factor-related protein 2) also contains PWWP and IBD domains, responsible for its interaction with the trimethylated H3K36me3 histone and IN, respectively [145,146]. This being said, if the decrease of the cellular LEDGF/p75 level diminished the efficiency of integration, in cells with low HRP2 levels, HIV-1 replication remained at the wild-type level. When both HRP2 and LEDGF/p75 were depleted, the level of integration into active transcription regions was lower compared with cells where LEDGF/p75 was depleted alone [145,146]. HRP2 appears to provide vestigial integration in LEDGF/p75 knockdown cells, though its efficiency is lower than in control cells [147]. Interestingly, HRP2 overexpression in LEDGF/p75 knockout cells restored HIV-1 replication and integration up to the wild-type level [147].

### 7.3. INI1

Historically, INI1 is the first identified partner of IN [55]. INI1 is a component of the SWI/SNF ATP-dependent chromatin remodeling complex [148]. Positive [149,150,151] as well as negative [152] effects of this protein on HIV-1 replication have been reported. Although INI1 may influence HIV-1 integration site selection in vitro, it remains unclear if it is involved in this process upon infection. It is suggested that INI1, as part of the SWI/SNF complex, favors targeted integration into the genome. SWI/SNF is responsible for nucleosome remodeling, thus facilitating the access of IN to cellular DNA [153].

Cellular proteins LEDGF/p75 and INI1 are able to form a stable complex with IN and viral DNA [154]. The authors formed a complex of LEDGF/p75, HIV-1 IN, and the INI1 IN-binding domain (INI1-IBD) and studied its interaction with a DNA substrate—a 40-mer terminal fragment of U5 LTR region (U5-DNA). The presence of INI1-IBD did not affect the binding of this DNA but completely inhibited 3′-processing reaction catalyzed by IN. The analysis of U5-DNA integration into a plasmid (an analog of cellular DNA in this system) showed that the efficiency of integration by the IN/LEDGF/p75/INI1-IBD complex was significantly lower than that by IN/LEDGF/p75 or by IN itself. The authors hypothesized that to complete integration, plasmid DNA previously needs to displace INI1-IBD from the complex of IN/LEDGF/p75/INI1-IBD with viral DNA to enable the formation of a functionally active complex IN/LEDGF/p75/U5-DNA/plasmid DNA. This hypothesis was confirmed by the results of studying the IN/LEDGF/p75/INI1-IBD/U5-DNA complex structure by cryoelectron microscopy, which showed that INI1-IBD occupies a channel in the structure of IN which is crucial for IN binding to cellular DNA and that it limits conformational mobility of the IN tetramer [154]. Possibly, INI1 and LEDGF/p75 together stabilize the multimer IN form to prevent unfavorable interactions of IN with other proteins as well as viral DNA autointegration when PIC is translocated from the cytoplasm to the integration site.

## 8. DNA-Repair Factors Interacting with Integrase

### 8.1. hRAD51 and hRAD18

The hRAD51 protein from the cellular homologous recombination DNA repair system directly interacts with HIV-1 IN in vitro and inhibits its catalytic activity both in vitro and in a yeast cellular integration system [155]. It was also demonstrated that the formation of an active hRAD51 nucleofilament is required for optimal inhibition through an IN-DNA complex dissociation mechanism. This inhibition could be promoted in HIV-infected cells by chemical stimulation of the endogenous hRAD51 protein [156]. While chemical activation of hRAD51 stimulated its capacity to inhibit integration, its inhibition increased the integration efficiency, indicating that the modulation of HIV-1 integration depends on the hRAD51 recombinase activity [157]. Of note, early stimulation of hRAD51 (before cell infection) affected integration negatively, whereas late stimulation exerted the opposite effect. According to the results obtained, hRAD51 performs different functions in HIV-1 integration regulation demonstrating an early inhibiting and a late stimulating effect [157].

The interaction of IN and the hRAD18 repair factor was revealed by a co-immunoprecipitation analysis [31]. It was shown that the hRAD18 fragment of residues 65-226 is sufficient for binding IN. The study also revealed IN and hRAD-18 co-localization in nuclear structures in co-transfected cells. Importantly, increased cellular levels of hRAD18 co-occurred with increased IN levels, which indicates that hRAD18 may stabilize IN in cells. However, to date, the details of the mechanism of this effect are not totally clear.

### 8.2. Ku70

The integration of the viral DNA into the cellular genome results in single-stranded 5-nucleotide gaps in the cellular DNA, which flank the integrated viral DNA, as well as in unpaired CA dinucleotides on the 5′ ends of the viral DNA. Further successful viral replication requires the repair of these DNA damages [158]. The mechanism of post-integration repair has been widely studied and numerous data indicate that it involves proteins from the NHEJ system (non-homologous end joining), which mediates double-stranded DNA breaks repair [23,159,160,161,162]. The key NHEJ component is the DNA-PK complex (DNA-dependent protein kinase) [163]. The double-stranded DNA break is recognized by the Ku70/Ku80 dimer, which is a component of DNA-PK, which recruits the catalytic subunit of the DNA-dependent protein kinase DNA-PKcs to the break site. This results in the formation of a heterotrimeric DNA-PK complex, after which the repair mechanism is triggered [163].

Integration does not result in double-stranded breaks and, to initiate post-integration repair, the Ku70 protein, which is a component of the NHEJ system, directly binds IN [22,23]. Upon Ku70 knockdown in infected cells, post-integration repair was significantly suppressed, but integration and reverse transcription were not [23]. Co-precipitation allowed identifying the binding sites of IN and Ku70: the α6-helix of IN (residues 200–220) binds the N-terminal domain of Ku70 (1–250) [22]. The study also demonstrated that residues E212 and L213 in the α6 helix of IN are crucial for the interaction between IN and Ku70. Ku70 residues involved in the IN binding are S69, I72, S73, I76 [164]. In the same study, a model of the IN-Ku70 complex was suggested and a drug inhibiting the complex formation was designed. The crucial role of IN-Ku70 binding for successful post-integration repair and viral replication is generally confirmed by the fact that both processes were disrupted in the case of pseudovirus carrying E212A and L213A substitutions in IN, which impair its ability to bind Ku70, but not its catalytic activity [23].

### 8.3. FANCI-D2 Complex

Recently, the cellular complex FANCI-D2 from the Fanconi anemia DNA repair pathway was identified as another functional partner of IN, that binds to its C-terminal domain [165]. Notably, two proteins in this complex FANCI and FANCD2 are able to interact with HIV-1 IN. The knock-out of the FANCI or FANCD2 in HEK293T cells using CRISPR-Cas9 decreased the expression of the reporter gene encoded in a single-round HIV-1 vector. However, overexpression of proteins in corresponding knock-out cells restored reporter expression. The observed effects were independent of the influence the FANCI or FANCD2 knock-out on cellular DNA replication as they were the same in non-dividing cells, in which the cell cycle was arrested by aphidicolin. At the same time, S. Fu et al. demonstrated the impact of FANCI-D2 complex components for efficient integration of viral cDNA, and no effect on the reverse transcription. Based on the cellular function of the FANCI-D2 complex and the fact that the knockout of DNA polymerases or Flap nuclease downstream of FANCI-D2 reduced the levels of integrated HIV-1 DNA, the author suggested these proteins may be responsible for the repair of DNA damages induced by viral DNA integration. However, a decrease in the amount of integrated DNA immediately after the start of integration (12 h.p.i) and an increase in the amount of unintegrated 2-LTR circles (24 h.p.i. or later) in FANCI and FANCD2 knock-out cells may indicate the influence of these proteins and downstream targets directly on the integration process rather than on post-integration DNA repair. To confirm one of the possible hypotheses, it will be necessary to measure the efficiency of post-integration DNA repair, e.g., using the qPCR assay described in [166].

## 9. Conclusions

Finishing the description of cell partners of HIV-1 IN, we would like to note two points. First of all, we do not pretend to describe all cell proteins that somehow interact with IN, there are a lot of them, and we could miss some proteins. However, we have tried to list those partners for which binding to IN has been experimentally proven at the level of recombinant proteins and in cell culture, and for which it has been shown that their interaction with IN does indeed affect HIV-1 replication (Table 1). The second most important isthe study of cellular proteins involved in the life cycle of HIV-1 is important not only for a better understanding of the pathogenesis of this dangerous infection but for developing new approaches to fight it. Unlike viral proteins, which rapidly mutate under the pressure of drug selection, host proteins are relatively unchanged; moreover, the number of mutations in viral proteins interacting with host cell proteins that would not disrupt this interaction is extremely limited. Accordingly, drugs targeting complexes of viral and cellular proteins will obviously provide a high genetic barrier to the development of drug-resistant strains of the virus [167].

Studies of HIV-1 cell partners have already led to the development of the drug maraviroc inhibiting HIV gp120 protein binding to the CCR5 receptor [168] and an inhibitor of viral capsid assembly-disassembly lenacapavir, which prevents the viral capsid protein (CA) from interacting correctly with cellular proteins CPSF6 (specificity factor subunit 6) and nucleoporin Nup153 [169]. As for IN partners, inhibitors of IN interaction with LEDGF/p75 have already been developed [128,170]. Unfortunately, the molecular mechanisms by which these compounds inhibit HIV replication have not been fully established, and some of them may inhibit post-integration events even more effectively than the integration process itself [171]. Active work is also underway to create inhibitors of IN binding to the Ku70 protein, which activates post-integration repair of the cell genome [164]. We hope that this review will be useful in finding new targets for antiretroviral therapy.

## Figures and Tables

**Table 1:** Effects of cellular proteins interacting with HIV-1 on viral replication.

Cellular Protein Name	Affected Stage of HIV-1 Replication	Influence on HIV-1 Replication
TRIM33	Reverse transcription	Negative
	Integration	Negative
VBP1	Viral transcription immediately after integration	Positive
VHL		
p300	Integration	Positive
GCN5	Data inconsistent: reverse transcription, integration, viral transcription immediately after integration	Positive
HDAC1	Data inconsistent: reverse transcription or integration	Data inconsistent
KAP1 (TRIM28)	Integration	Negative
HDAC10	Integration	Negative
JNK and Pin1	Integration	Positive
GCN2	Integration	Negative
UBC9	Data inconsistent: integration or/and morphogenesis	Data inconsistent
SUMO3	Unclear	Unclear
Importin α (Imp α3)	Nuclear import	Positive
Importin 7	Nuclear import	Positive
Transportin-SR2	Nuclear import	Positive effect on PIC nuclear import, however this effect may be explained by Transportin-SR2 interaction with capsid protein rather than integrase
NUP153	Nuclear import	Positive
LEDGF/p75	Integration site selection	Positive
HRP2	Integration site selection	Positive when LEDGF/p75 expression inhibited No effect under normal LEDGF/p75 level
INI1	Unknown (Integration site selection?)	Data inconsistent
hRAD51	Integration	Complex: activation of hRad51 before infection reduces integration, the late activation of hRad51 activity stimulates integration
hRAD18	Unknown	Unknown
Ku70	Post-integration DNA repair	Positive
FANCI	Integration or post-integration DNA repair	Positive
FANCD2	Integration or post-integration DNA repair	Positive
